# Optimization of Heat-Dissipation Structure of High-Power Diode Laser in Space Environments

**DOI:** 10.3390/mi15080968

**Published:** 2024-07-29

**Authors:** Lei Cheng, Huaqing Sun, Xuanjun Dai, Bingxing Wei

**Affiliations:** 1 College of Mechanical and Control Engineering, Guilin University of Technology, Guilin 541000, China; sunhuaqing@jcgjd.org.cn (H.S.); weibingxing2023@163.com (B.W.); 2 Jincheng Research Institute of Opto-Mechatronics Industry, Jincheng 048000, China; 3 Shanxi Key Laboratory of Advanced Semiconductor Optoelectronic Devices and Integrated Systems, Jincheng 048000, China

**Keywords:** space environment, high-power laser diode, heat dissipation, microchannel heat sink, structure optimization

## Abstract

The high-power laser diode (HPLD) has witnessed increasing application in space, as the aerospace industry is developing rapidly. To cope with the space environment, optimizing the heat-dissipation structure and improving the heat-dissipation ability via heat conduction have become key to researching the thermal reliability of the HPLD in space environments. Based on a theoretical analysis of the HPLD, a simulation model of the HPLD was constructed for numerical simulation, and it was found that the maximum temperature and thermal resistance of lasers were efficaciously decreased by changing the packaging position of laser bars. The packaging position of the bars and the cutting angle of the microchannel heat sink (MCHS) were determined based on the light-emitting angle of the light-emitting unit and the internal structure of the MCHS. The internal structure of the MCHS was optimized through a single-factor experiment, an orthogonal experiment, and the combination of neural networks and genetic algorithms (GAs), using three key structural parameters, namely the MCHS ridge width, W1, the channel width, W2, and the channel length, L1. After optimization, the performance of the MCHS was obviously improved. Finally, an analysis was carried out on the applicability of the optimized MCHS to bars with a higher power.

## 1. Introduction

The laser diode (LD) has shown rapid development since 1962, when the world’s first LD came into being, with increasing types and expanding application scope [1,2,3,4,5]. As a class of laser-generation devices with semiconductor materials as the operating substance, LDs have gradually become one of the indispensable photoelectric devices in modern science and technology, following decades of development. The HPLD, owing to small volume and mass, high electro-optical conversion efficiency, long service life and wide wavelength coverage range, have been extensively applied in fields such as industry, the military, communication, optical storage and laser medicine in recent years [6,7]. LD has electro-optical conversion efficiency as high as about 50%. Nowadays, the highest electro-optical conversion efficiency of the HPLD merely reaches 76% [8,9,10], which was achieved by careful optimization of quantum well properties using an improved single-stripe laser device at a temperature of 10 °C and a wavelength of 975 nm. This means that a considerable amount of electrical energy will be converted into heat. Besides, HPLD is characterized by very concentrated heat generation, enabling the generation of heat in extremely high heat flux per unit volume. Therefore, the laser chip will experience temperature increment in the case of a failure to timely conduct out a great deal of heat generated during operation, which will give rise to a red shift in the laser-emitted wavelength, unstable output power, increased threshold current, mode jump, and internal defects, further inducing catastrophic optical damage and even resulting in the burnout of lasers. According to investigations on service environment of photoelectric devices, temperature variation is responsible for 55% of their faults [11]. Reliable packaging of the LD can ensure that the chip works in a relatively stable environment, enhancing heat-dissipation performance, prolonging the operating life and realizing easy transportation and installation. Thence, research hotspots have lain in how to enhance the heat-dissipation capacity of the HPLD packaging, effectively conduct out the heat generated during operation and improve the thermal reliability of the HPLD.

**Figure 1:**
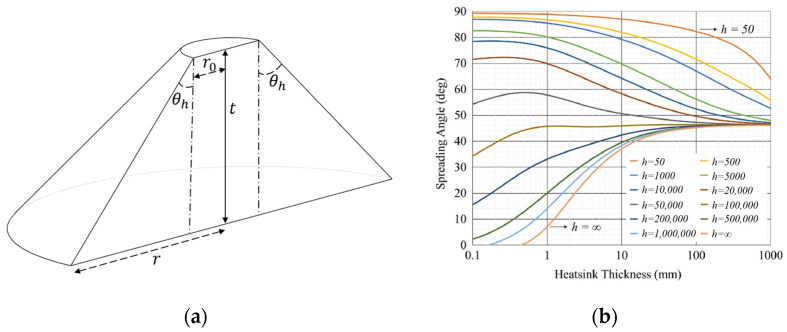
Heat conduction direction. (**a**) Schematic diagram of heat flow conduction in heat sinks at a fixed angle. (**b**) the study of heat diffusion angle [20].

HPLDs realize heat control mainly through heat convection, heat radiation, and heat conduction. With advances in technologies such as inter-satellite laser communication and laser radar, the HPLD has displayed an expanding application in space, but heat convection, a crucial heat-dissipation approach on earth, becomes almost ineffective in space. In space environments, heat conduction serves as the major heat-dissipation approach of the HPLD [12,13]. Currently, common heat conduction approaches for heat dissipation [14] include heat-sink conduction cooling [15], large-channel water cooling [16], microchannel water cooling [17], spray cooling [18], and jet impingement [19]. Heat dissipation through the MCHS has become a preferable option considering the limitation of aeronautical environments, the heat-dissipation requirement of the HPLD, and the weight requirement of the heat-dissipation structure. The major factors affecting the heat dissipation of the microchannel structure include the position of laser bars and the key parameters designed for the MCHS. Di-Hai Wu et al. [20] introduced the concept of heat diffusion angle in engineering thermodynamics into the design of the heat sink, assuming that the heat flow was conducted from the top surface to the subsurface of the heat sink at a fixed angle $\theta_{h}$, and the effective heat flow section in the heat sink was semicircular. As shown in Figure 1a, $r_{0}$ stands for the equivalent radius of heat source, and t represents the thickness of the heat sink. The results showed that with the increase in the thickness of the heat sink, the heat flow always experiences further diffusion, and the effective heat diffusion angle approaches 45° when the thickness of the heat sink approaches infinity, as shown in Figure 1b. In Figure 1b, h is the convective heat transfer coefficient, expressed in W/m^2^K. It is evident from Figure 1 that in an ideal state, the heat generated by laser bars spreads downward at an angle of 45°, with the bar as the center. In engineering practice, the packaging position of bars is usually determined at the front end of the heat sink to reduce the packaging cost, which is feasible in the heat dissipation of low-power LDs. For the HPLD, however, forward diffusion of heat flow is unavailable, greatly increasing the thermal resistance of such lasers and affecting their thermal reliability. Wu et al. [21] studied the thermal characteristics of a microchannel-cooled high-power semiconductor laser array by a numerical method combined with a finite element method and computational fluid dynamics (CFD). They explored the steady-state and transient thermal behaviors of the device at different water flow rates in continuous wave mode in detail, analyzed the relationship between thermal resistance and water flow rate, and derived the thermal time constant to characterize the different heating processes of each part of the device. Shayan Pourhammati et al. [22] conducted numerical investigations on the fluid flow and heat transfer in the wavy MCHS via CFD, and sought out the desirable wavy channels by changing the relative values of wavelength and amplitude in the MCHS, which further improved the heat-dissipation performance of the wavy microchannel system. Zhou et al. [23] designed the microchannel configuration in the manifold microchannel heat sink (MMCHS) using a topology optimization method and proposed a manifold microchannel heat sink with a topologically optimized microchannel substrate (MMCHS-TOMS). With a volumetric flow rate of 1 L/min and a heat flux of 687 W/cm^2^, the MMCHS-TOMS achieved a maximum cooling coefficient of performance of 78,273, which is the highest value reported up to date. Existing research on the MCHS packaging has mostly focused on the internal structure, including but not limited to the optimization of the ridge width, channel length and width, and the enhancement of the convective heat transfer capacity of the MCHS and heat-dissipation performance of lasers. However, univariate analysis is employed in most studies on the internal structure parameters of the MCHS, and the impact of multi-factor interaction on the heat dissipation of the MCHS is not considered.

In this work, a three-dimensional physical model of the LD packaged on the MCHS was constructed, the heat-dissipation process of the MCHS was numerically simulated, and the influence rule of the packaging position of laser bars on the MCHS on heat transfer performance was explored. On this basis, a single-factor experiment was implemented to uncover the influences of key parameters (including ridge width, channel width and length) designed for the MCHS on heat-dissipation performance, and an orthogonal experiment with multivariate regression analysis was carried out on the interactive relationship between these key parameters. Moreover, a neural network was combined with the GA for multi-objective optimization, so as to obtain the optimal solution of key parameters designed for the MCHS.

## 2. Numerical Model and Theoretical Analysis

### 2.1. Physical Geometric Model

**Figure 2:**
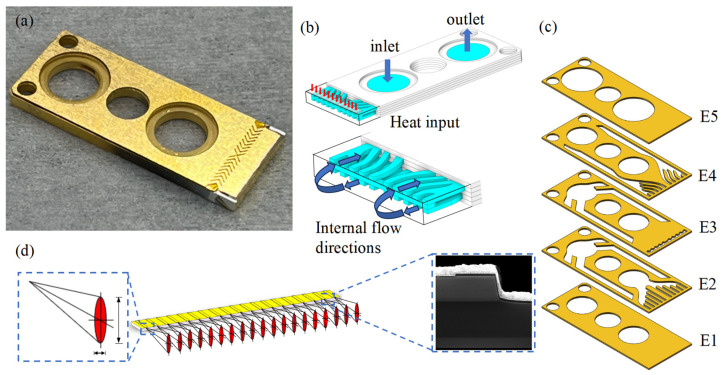
Schematic diagram of the LD. (**a**) Physical diagram of the LD in the MCHS package. (**b**) Schematic diagram of the MCHS heat dissipation. (**c**) Internal structure of the MCHS: E1, E2, E3, E4, and E5 are the bottom layer, inlet layer, separating layer, outlet layer, and top layer, respectively. (**d**) The LD bar and its local magnification.

As shown in Figure 2a, lasers were equipped with a microchannel water-cooled packaging structure, which consisted of an MCHS, a solder layer, an LD bar, an insulating film, and a copper sheet. The bar was packaged with the P surface facing downward and welded at the front end of the microchannel water-cooled heat sink with solder in the assembly process. Distilled water flowed into the internal MCHS from the water inlet, fully exchanged heat with the copper wall, took away the heat generated by the bar during the operation, and finally flowed out from the water outlet, as shown in Figure 2b. The use of water as a working medium in the space environment is feasible and has been applied. For example, the International Space Station uses water as the operating medium for the internal active thermal control system. The parameter of the MCHS was 27 mm × 10.8 mm × 1.5 mm, and the MCHS was composed of five layers of stitch-welded oxygen-free high-conductivity copper, as shown in Figure 2c. The LD bar studied in this paper had a bar wavelength of 808 nm, a ridge width of 150 μm, a filling factor of 30%, a cavity length of 1000 μm and 19 light-emitting points. At 25 °C, the injection current was 50 A, the maximum output power was 55.31 W, the electro-optical conversion efficiency was 58.74%, the slope efficiency was 1.21 W/A, and the divergence angles of the slow and fast axes of the laser die were less than 10° and less than 39°, respectively, as shown in Figure 2d. The data used in the calculation in this paper are shown in Table 1.

### 2.2. Mathematical Analysis on Heat Generation and Electro-Optical Conversion Efficiency

When the LD is operating, a lot of heat will be generated by the active layer, which, if not conducted out in time, will directly affect the operating characteristics, seriously affect the stability, and shorten the service life of the LD. The heat generated during the operation of the LD mainly comes from two aspects [24,25,26]:

(1) In the case of normal operation of the LD, the fairly large carrier density and photon density are found in the active region, which will trigger massive non-radiative recombination, radiation absorption, and spontaneous radiation absorption, thus generating a lot of heat. The joule heat generated in the active region was calculated as per the following equation [26]:
  (1) $$Q_{1}=\frac{V_{j}}{d_{active}}\left[j_{th}\left(1-\eta_{sp}f_{sp}\right)+\left(j-j_{th}\right)\times \left(1-\eta_{ex}\right)-\left(1-\eta_{i}\right)\eta_{sp}f_{sp}\right]$$
  where $Q_{1}$ is the heat power density in the active region, ${\;V}_{j}$ stands for the PN junction voltage, $d_{active}$ indicates the thickness of the active region, $j_{th}$ is the threshold current density of the laser, $\eta_{sp}$ represents the internal quantum efficiency of spontaneous radiation, $f_{sp}$ stands for the escape factor of spontaneous radiation photons from the active region, j is the injection current density, $\eta_{ex}$ indicates the external differential quantum efficiency (characteristic temperature: $T_{1}$), and $\eta_{i}$ represents the internal quantum efficiency of stimulated radiation.
(2) The following equation was adopted to compute the joule heat generated by semiconductor materials and ohmic contact resistance outside the active region [26]:
  (2) $$Q_{2}=j^{2}\rho$$
  where $Q_{2}$ and ρ correspond to the heat power density and electrical resistivity of materials outside the active region.

According to the heat generation mechanism of the LD described above, more heat will be generated due to various energy losses during the normal operation of such lasers, and an excessively high temperature will have a great impact on the performance and reliability of the LD [27,28]. The output power decreases significantly with the increase in temperature. The relationship between the output power and temperature is determined as follows [27]:

(3) $${P=\eta }_{slope}\left(T\right)\left(I-I_{th}\left(T\right)\right)$$

where $\eta_{slope}\left(T\right)$ is the optical power-slope efficiency of operating current, $I_{th}\left(T\right)$ represents the threshold current, and I stands for input current.

When the operating current was greater than the threshold current, the output power rose linearly with the operating current. The change in slope efficiency with the change in temperature was expressed as follows [27]:

(4) $$\eta_{slope}\left(T\right)=\eta_{Ref}e^{-\left(\frac{T-T_{Ref}}{T_{0}}\right)}$$

where $\eta_{Ref}$ is the slope efficiency at the reference temperature, $T_{Ref}$ is the reference temperature, and $T_{0}$ is the characteristic temperature for the threshold current.

The increase in temperature will lead to a change in the gain coefficient, an increase in the loss coefficient, and a continuous decrease in internal quantum efficiency, thereby increasing the threshold current. The empirical equation of the relationship between threshold current and temperature is presented below [27]:

(5) $$I_{th}\left(T\right)=I_{Ref}e^{\frac{T-T_{Ref}}{T_{0}}}$$

where $I_{Ref}$ is the threshold current at the reference temperature [29,30].

Combined with Equations (3)–(5), the following relationship between output power and temperature is established:

(6) $${P=\eta }_{Ref}e^{-\left(\frac{T-T_{Ref}}{T_{0}}\right)}\left(I-I_{Ref}e^{\frac{T-T_{Ref}}{T_{0}}}\right)$$

Because temperature has a great influence on the output power of semiconductor lasers, temperature also exerts a similar effect on the electro-optical conversion efficiency. The relationship between temperature and electro-optical conversion efficiency was expressed as follows [30]:

(7) $$\eta_{P}=\frac{P}{IV+I^{2}r}$$

where V and r are the forward bias value and the sum of the electrical resistivity of materials outside the active region, respectively.

## 3. Analysis on the Influence of Bar Packaging Position on Heat Dissipation of the LD

### 3.1. Numerical Analysis

**Figure 3:**
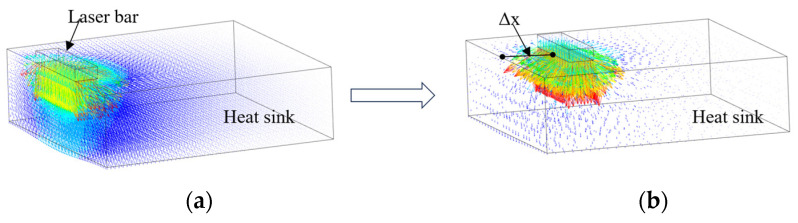
A schematic diagram of heat flow conduction in heat sink after the LD bar generates heat. (**a**) The bar is in the original package state, and the heat flow can only be conducted downward and backward in large quantities. (**b**) The LD bar packaging position moved back a certain distance; at this time, the heat generated by the LD bar can be diffused and conducted around, which facilitates the transfer of the bar heat and reduces the temperature of the LD bar.

As previously mentioned, when the packaging position of the LD bar was parallel to the front end surface and on the same plane of the MCHS, the heat near the light-emitting surface of the chip could only be transferred downwards in large quantities, as shown in Figure 3a. After the rearward shift of the bar, the heat could be transferred to many directions because the MCHS is substantially larger than the bar in terms of the longitudinal dimension, as shown in Figure 3b, with Δx denoting the rearward shift distance of the bar. To improve the thermal reliability of the HPLD, it is of great significance to determine the rearward shift distance of the bar and explore the influence rule of bar packaging position on the heat dissipation of the HPLD. A fluid–solid coupled conjugate heat transfer model of the HPLD was established using finite element software. To reduce the calculation amount and improve the calculation speed, the LD was simplified reasonably without affecting the simulation results, and only the structures such as the LD bar and MCHS were retained, whereas other structures with little influences on heat dissipation were ignored. Combined with the space environment, the condition was set without heat convection. As to the choice of models, the realizable k−ε model and the surface-to-surface model were selected as the turbulence calculation model and radiation model, respectively. The coolant inside the MCHS was driven by an external pump, with the inlet water flow rate and water temperature set at 6 m/s and 25 °C, respectively. Since the outlet pressure and velocity were unknown, the outlet was set as free outflow. Meshing by mapping mode was implemented, and mesh encryption was applied to the bar, lower interconnection interface, and other key regions of concern. The mesh size was 1 × 10^−4^ m, and that of the heat sink was 5 × 10^−4^ m. The whole LD bar was considered as a heating region. It is assumed that the volume heat generation rate of the whole semiconductor laser is uniform. According to the following calculation formula, the heat generation rate is 2.59 × 10^10^ W/m^3^.

(8) $$H=\frac{Q}{V}=\frac{P_{out}\left(\frac{1-\eta }{\eta }\right)}{V}$$

where Q is the waste heat generated by the LD bar, V is the volume of active layer of the LD bar, which is simplified to the entire laser bar in this paper, $P_{out}$ is the output power of the LD bar, and η is the electro-optical conversion efficiency of the LD bar.

The influence of bar rearward shift distance Δx at an interval of 0.5 mm on the heat-dissipation performance of the LD is discussed.

### 3.2. Results and Discussion

**Figure 4:**
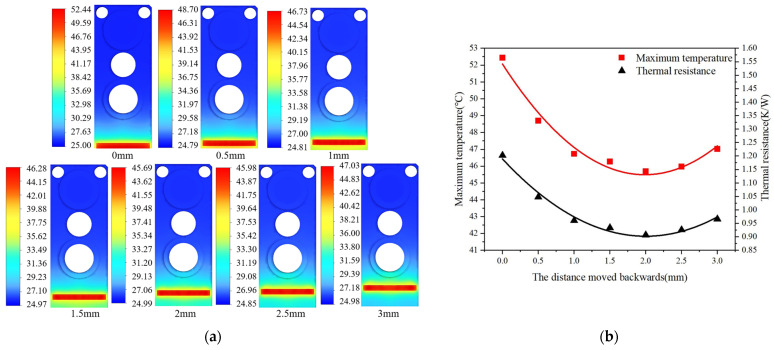
Influence of bar rearward shift by different distances on temperature of semiconductor lasers. (**a**) Nephogram of temperature distribution of packaging structure at different bar rearward shift distances. (**b**) Fitting curves of maximum temperature and thermal resistance of the bar at different packaging positions.

Figure 4a is the nephogram of temperature distribution at different LD bar rearward shift distances. It can be seen that the heat was gradually transferred to many directions, as the LD bar moved rearward gradually at an interval of 0.5 mm from the position parallel to the front end surface and on the same plane of the MCHS. When the rearward shift distance was far enough, the heat was transferred to the surroundings and lower part, with the LD bar as the symmetry axis. The fitting curves of the maximum temperature and the thermal resistance of the LD bar at different packaging positions are shown in Figure 4b, where the X-axis is the LD bar rearward shift distance of (0–3 mm), and the left Y-axis and right Y-axis are the maximum temperature and thermal resistance of the LD at different bar rearward shift distances, respectively. The calculation formula of thermal resistance is as follows [31]:

(9) $$R_{th}=\frac{T_{1}-T_{2}}{P_{th}}$$

where $T_{1}$ is the working junction temperature of the LD bar, $T_{2}$ is the initial temperature of the LD, and $P_{th}$ is the thermal power of the LD bar.

**Figure 5:**
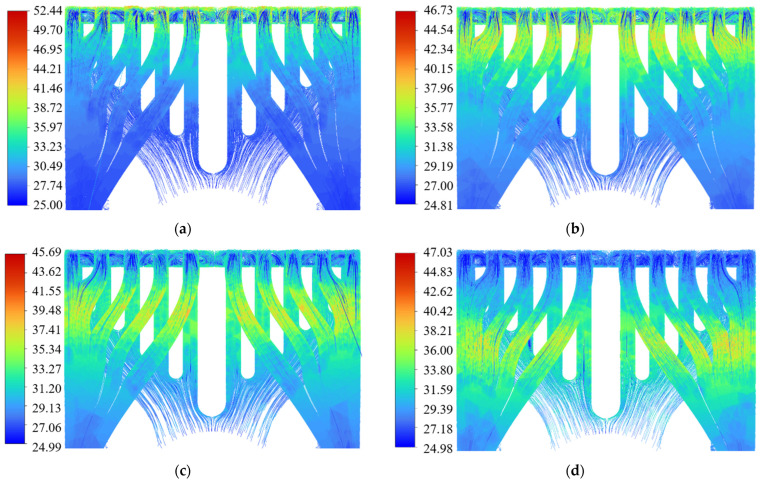
Trace diagram of bar rearward shift by different distances of 0 mm (**a**); 1 mm (**b**); 2 mm (**c**); and 3 mm (**d**).

In the range of 0–2 mm, the temperature of the chip dropped significantly with the increase in the rearward shift distance. When the LD bar rearward shift distance reached 2 mm, the maximum temperature and thermal resistance of the LD declined by 6.75 °C and 24.6%, respectively, compared to those before rearward shift, suggesting an improvement in the overall heat-dissipation performance of the LD. However, when the LD bar rearward shift distance exceeded 2 mm, the maximum temperature of the laser started to rise again, which is mainly attributed to the effect of the MCHS structure. Figure 5 is the trace diagram for the thermal simulation results at the LD bar rearward shift distances of 0 mm, 1 mm, 2 mm, and 3 mm. Apparently, when the LD bar rearward shift distance reached 2 mm, the effective conduction area of the LD bar temperature to the water flow region was larger than that at the bar rearward shift distances of 0 mm, 1 mm, and 3 mm. When the LD bar rearward shift distance was 0 mm, the heat generated by the bar could only be conducted downwards and rearwards. In the case of the LD bar rearward shift distance of 2 mm, the heat generated by the bar could be conducted forwards, as well as downward and rearward. However, when the LD bar rearward shift distance reached 3 mm, the heat-dissipation capacity decreased along with the rearward conduction of the heat generated by the LD bar due to the shrinkage of the fluid channel and changes in the internal structure of the MCHS. Hence, it is evident that the maximum temperature would rise continuously if the LD bar continued to move rearwards.

## 4. Analysis and Optimization of Key Parameters of the MCHS

**Figure 6:**
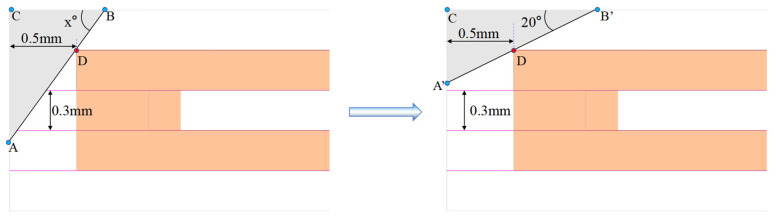
Schematic diagram of the MCHS affecting the cutting of the light-emitting part.

According to the research described above, the temperature peaked near the light-emitting surface of the chip, where heat dissipation was worst, before the rearward shift of the LD bar. Through the LD bar rearward shift, the overall heat-dissipation performance of the LD, as well as the heat-dissipation capacity near the light-emitting surface, is enhanced, but the light-emitting path in the front cavity is blocked [32]. It is a simple and feasible method to cut the part that blocks the light. When studying the cutting angle and cutting distance, it must be first ensured that it will not affect the microchannel structure. That is, the gray part (cutting part) and the orange part (microchannel heat sink internal channel structure) in the figure do not overlap. Combined with the fact that the above-mentioned laser bar’s fast axis divergence angle is less than 39°, the cutting angle must be greater than or equal to 20°. Otherwise, it will still affect the light. Combined with Figure 1a, it can be seen that when the cutting angle is greater than 45°, the heat flow diffusion path is obstructed, so the cutting angle is better in the range of 20–45°. Cutting research is carried out in a limiting way (the gray part just touches the orange part and intersects at point D). It can be clearly seen in the figure that as the cutting angle x (x > 20) decreases, the distance (BC) that the bar can move backward increases, which can be simplified as a straight line AB rotating around point D. When the cutting angle’s limit is 20°, the backward distance reaches the maximum (B’C) of 1.324 mm. Combined with Figure 4b, it can be seen that within the range of 0–2 mm retreat, the maximum temperature shows a downward trend with the increase in the retreat distance. In summary, the cutting angle of 20°is a better choice, as shown in Figure 6.

To ensure the reliability of the MCHS under hydraulic impact, the rearward shift distance of 1 mm was selected in this work, and a safety distance of 0.324 mm was reserved. At this point, the minimum thickness of the wall in the MCHS was calculated as 0.1 mm. To prevent the MCHS from damage by hydraulic pressure, a copper tube with a wall thickness of 0.1 mm and an outer diameter of 10 mm was constructed for the computation of pressure-bearing capacity. The formula used for reference is applied to seamless copper water pipes and copper gas pipes. This formula is combined with the empirical formula obtained [33]:

(10) $$P=\frac{2S\times t\times \varphi \times A}{D-0.8t}$$

where P is the maximum operating pressure in MPa, S is the allowable stress of materials, which was 67 MPa in this study, and D stands for the outer diameter of the copper tube; t is the wall thickness, φ represents the welding coefficient, which was 0.8 in this study, and A is the copper tube stretching compensation, which was 2/3 in this study. The calculated result was 0.72 MPa, signifying that the maximum hydraulic pressure cannot exceed 0.72 MPa.

**Figure 7:**
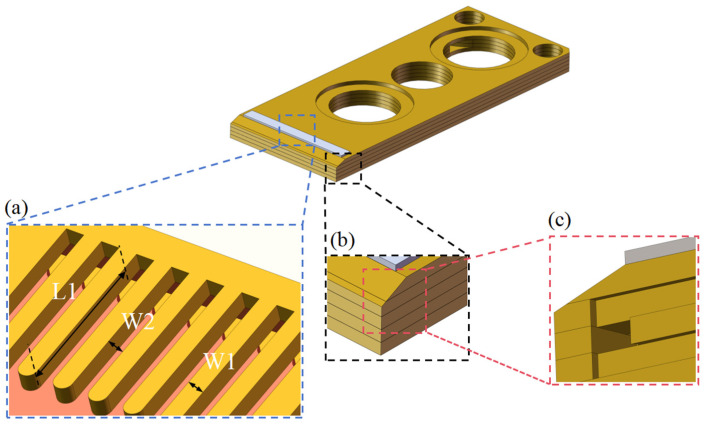
Optimization diagram of the MCHS. (**a**) Optimization diagram of each parameter. (**b**) Partial magnification. (**c**) Internal diagram of the partial amplification section.

Moreover, key parameters designed for the MCHS, namely a ridge width W1 between channels, channel width W2, and channel length L1, were selected for an influence study, as shown in Figure 7. The ranges of W1, W2, and L1 were 0.1–0.5 mm, 0.1–0.5 mm, and 0–4 mm, respectively. In this study, W1 and W2 were independent of each other. Changing either W1 or W2 would give rise to variations in the number of microchannels and fins.

### 4.1. Influence of Different Structural Parameters

#### 4.1.1. Influence of Parameter W1

To explore the influence of W1 between channels on the MCHS, five groups of inter-channel ridge widths, namely, 0.1 mm, 0.2 mm, 0.3 mm, 0.4 mm, and 0.5 mm, were set for the simulation study on the maximum temperature and pressure of the HPLD, with W2 and L1 valued at 0.3 mm and 2 mm, respectively.

**Figure 8:**
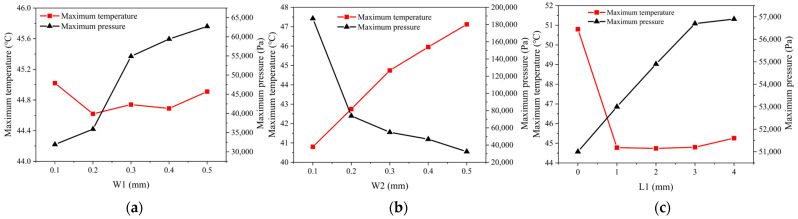
Influence of parameters on maximum temperature and maximum pressure of the MCHS. (**a**) Effects of W1 on the maximum temperature and pressure of the MCHS. (**b**) Effects of W2 on the maximum temperature and pressure of the MCHS. (**c**) Effects of L1 on the maximum temperature and pressure of the MCHS.

As shown in Figure 8a, the maximum temperature of the HPLD declined when W1 changed from 0.1 mm to 0.2 mm and fluctuated slightly when W1 changed from 0.2 mm to 0.5 mm. The upward trend of the maximum pressure in the MCHS gradually became stable, and the maximum pressure was 0.0627 MPa at 0.5 mm, which is far less than the safe value of 0.72 MPa. In the case of an increase in W1, the effective heat conduction area of the outlet layer and top layer was increased, but the number of microchannels was reduced, and the convection area between the microchannel wall and the coolant liquid changed, resulting in fluctuations in temperature.

#### 4.1.2. Influence of Parameter W2

In addition, a simulation study was also implemented on the maximum temperature and pressure of the HPLD at five groups of different microchannel widths, namely, 0.1 mm, 0.2 mm, 0.3 mm, 0.4 mm, and 0.5 mm under W1 at 0.3 mm and L1 at 2 mm, so as to explore the influence of the microchannel width W2 on the HPLD.

According to Figure 8b, when W2 changed from 0.1 mm to 0.5 mm, the maximum temperature of the HPLD rose almost in a linear manner, with a slope of 15 °C/mm. The maximum pressure of the MCHS decreased rapidly and then tended to be stable, which peaked (0.187 MPa) at 0.1 mm. This value is far lower than the safe value of 0.72 MPa. The variation in W2 led to the change in the convective area between microchannel wall and coolant liquid, and it can be concluded that there is an almost linear correlation between the maximum temperature and the convective heat transfer area between the microchannel wall and the coolant liquid. An appropriate increment in W2 has a significant effect on reducing the maximum pressure of the MCHS.

#### 4.1.3. Influence of Parameter L1

To probe into the influence of L1 on the HPLD, five different groups of microchannel lengths, namely, 0 mm, 1 mm, 2 mm, 3 mm, and 4 mm were set for a simulation study on the maximum temperature and pressure of the HPLD under the premise of 0.3 mm W1 and 0.3 mm W2.

As shown in Figure 8c, the maximum temperature dropped rapidly when L1 rose from 0 mm to 1 mm, while it fluctuated slightly at 1–4 mm. An elevation in L1 could increase the effective heat conduction area of the outlet’s layer and top layer, as well as the convective heat transfer area between the microchannel wall and the coolant liquid. When L1 was greater than 1 mm, the heat transfer approached an equilibrium state. The maximum pressure of the HPLD rose rapidly and then tended to be stable, peaking (0.0569 MPa) at 4 mm. This value is far lower than the safe value (0.72 MPa).

The above analyses on the influences of single microchannel structural parameters on the LD temperature and pressure were completed. The safety value of the MCHS pressure was 0.72 MPa. This value is much greater than the maximum pressure in the above univariate analysis. Therefore, when the water flow rate is 6 m/s, the pressure generated by water flow does not have a destructive effect on the MCHS. Consequently, the influence of pressure on the MCHS will not be considered in subsequent sections.

### 4.2. Design of Orthogonal Experiment and Establishment of Regression Equation

To analyze the comprehensive influence of three structural factors (W1, W2, and L1) on the heat-dissipation performance of the LD, an orthogonal experiment was conducted to obtain the regression equation, and regression equation fitting was then accomplished to obtain the functional relationship between the structural parameters and the maximum temperature.

Through the above analysis, three factors, namely, W1, W2, and L1, were selected in this paper. Within their respective value ranges, three levels were taken, namely, the minimum, median, and maximum. The levels of influencing factors are listed in Table 2. The orthogonal experiment table was constructed by Design-Expert. Based on this table, the simulation experiment was carried out with the values of various influencing factors as independent variables and the maximum temperature as the evaluation index. The test results are shown in Table 3.

According to the data samples in Table 3, the quadratic polynomial regression model of the maximum temperature was obtained:

(11) $$Y=44.68-0.51W1+3.42W2-2.53L1-1.70W1W2+0.73W1L1-0.21W2L1+0.38{W1}^{2}-0.74{W2}^{2}+2.87{L1}^{2}$$

Next, goodness-of-fit R2 analysis was implemented on the quadratic polynomial regression model, and it was uncovered that the closer the value of goodness-of-fit R2 was to 1, the higher the credibility of the model would be. The goodness-of-fit R2 of the regression model for the maximum temperature of the MCHS was 0.9907, suggesting that this model is reliable.

### 4.3. Optimization Solution through Combination of Neural Network Algorithm and GA

Both neural network algorithms and GA are crucial theoretical achievements formed by simulating laws in nature [34,35,36,37]. The former improve performance by constantly adjusting the structure and parameters of the neural network, whereas the latter searches for the optimal solution by simulating the biological evolution process. The combination of the two can not only take full advantage of the learning ability of the neural network but also further enhance the performance through the optimization of GA.

The main process of combining a neural network algorithm and GA to optimize the solution is to first input the training sample into the neural network model and conduct some learning and training on the model. The model, trained to the required accuracy, will be used to predict the output value of the system, that is, to predict the maximum temperature of the HPLD. Then, the selected groups of predicted data samples are input into the trained neural network model to verify the accuracy of the model prediction. The obtained accurate prediction model function is passed into the GA model structure as the objective function, and the sample input parameter combination is taken as the optimization objective. Through the iterative calculation of the genetic algorithm, the optimal target parameter combination is finally obtained, and the prediction of the maximum temperature and the optimization of the parameter combination are completed.

In this paper, the Levenberg–Marquardt algorithm is chosen as the learning algorithm for the neural network. In this paper, four parameters, including W1, W2, L1, and flow rate (V) are mainly considered to predict the maximum temperature of the LD. Therefore, there are four nodes in the input layer and one node in the output layer. There is no universal and accurate method for determining the number of nodes in the hidden layer, but the following empirical formulas can be referred to [38]:

(12) $$h>\frac{m+n}{2}$$

(13) $$h=\sqrt{m+n}+a$$

(14) $$h=\log_{2}m$$

(15) h=2m+1

where m is the number of nodes in the input layer, n is the number of nodes in the output layer, h is the number of nodes in the hidden layer, and a is a constant between 1 and 10.

After training the neural network with different numbers of hidden layer nodes, it was observed that the more the number of hidden layer nodes, the smaller the error, and the number of hidden layer nodes was finally selected as 10.

The structural parameters of the GA are determined. According to the above analysis, the highest temperature of the laser chip is the evaluation criterion, and the optimal design objective function is as follows:

(16) $$\left\{\begin{array}{c} \min\left[F(X)=f(x_{i1},x_{i2},x_{i3},x_{i4})\right]\\ X=\left[X_{11},X_{12},X_{13},X_{14}\ldots \ldots ,X_{n1},X_{n2}{,X}_{n3}{,X}_{n4}\right]\\ s.t\;0.1\leq X_{i1}\leq 0.5,\;0.1\leq X_{i2}\leq 0.5,\;0\leq X_{i3}\leq 4,\;2\leq X_{i4}\leq 6\; \end{array}\right.$$

where $f(x_{i1},x_{i2},x_{i3},x_{i4})$ is the maximum temperature function of the LD chip output by the neural network, and $x_{i1},x_{i2},x_{i3},x_{i4}$ are the four factors of the sample. $0.1\leq X_{i1}\leq 0.5,0.1\leq X_{i2}\leq 0.5,0\leq X_{i3}\leq 4,2\leq X_{i4}\leq 6$, respectively, represent the feasible ranges for the corresponding factors. Set the population size to 50 and the evolutionary algebra to 200. Keep the default constraint dependent for crossover and mutation operations.

**Figure 9:**
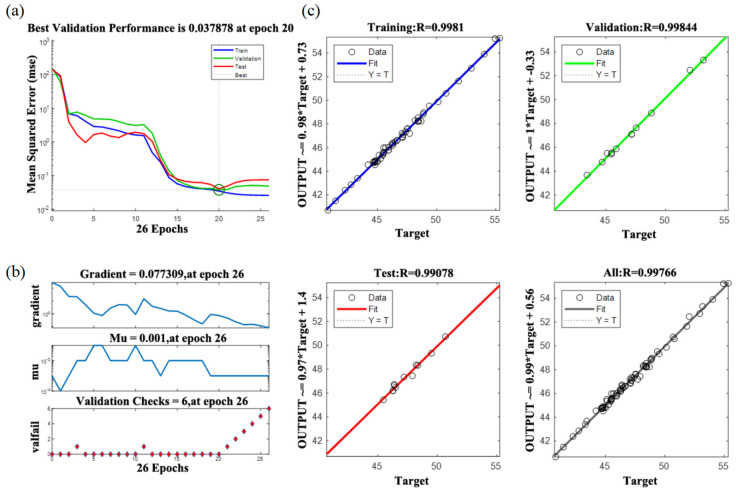
The model training results. (**a**) Neural network training error MSE results; (**b**) neural network training results; and (**c**) neural network model fitting results.

In this study, 80 groups of experimental data were used for the experiment in the MATLAB 2018 environment. The model training results are shown in Figure 9.

To verify the accuracy of the training results, eight groups of parameter combinations were randomly designed within the parameter ranges, and then the training results (R1) were obtained. The corresponding test results (R2) were calculated by the thermal simulation analysis model. Relevant data are stated in Table 4.

**Figure 10:**
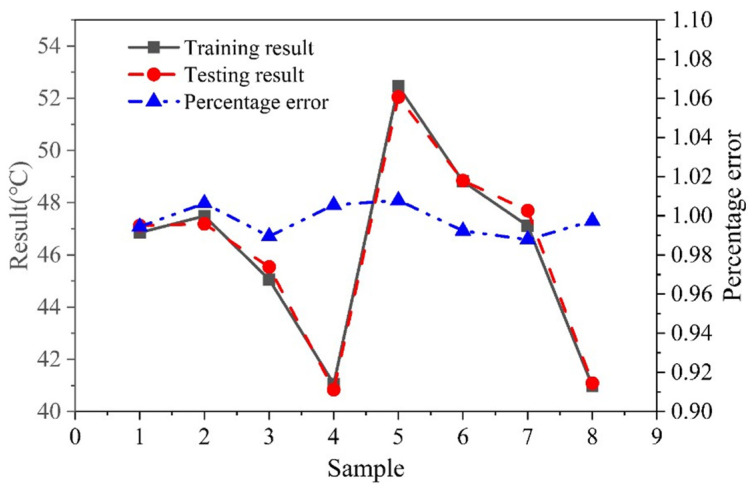
Training results and test results, together with corresponding error curves. The blue dots represent the percentage error between the training results and the test results.

The relation curve of the maximum temperature and the corresponding error curve, as shown in Figure 10, were plotted based on the data from the training results and test results shown in Table 2. According to Figure 9, the training results of eight random samples were very close to the test results, and the maximum error of the maximum temperature was 1.2%, within the range of 5%. The model accurately reflected the nonlinear problems of the actual operating conditions, so the neural network model could be utilized as a mathematical model for predicting the maximum laser temperature.

**Figure 11:**
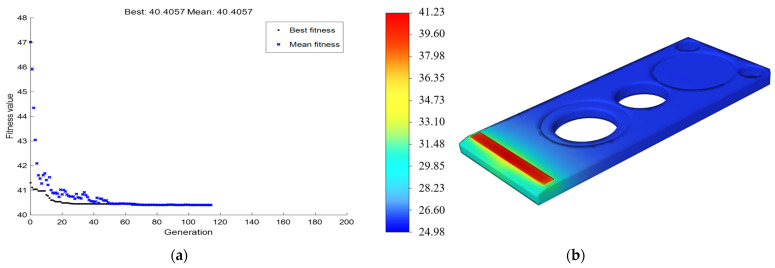
Optimal parameter combination. (**a**) The optimal result was obtained by combining a neural network and GA, and the maximum temperature was 40.4057 °C. (**b**) The optimal parameter combination test results. The highest temperature is 41.23 °C.

Thereafter, a GA optimization model was established based on the above model, and the optimal parameter combination obtained by repeated optimization was as follows: ridge width W1 = 0.5 mm, microchannel width W2 = 0.1 mm, microchannel length L1 = 1.219 mm, and maximum temperature = 40.4057 °C, as shown in Figure 11a. An MCHS model was then constructed with the optimal parameter combination, and the test results were obtained through numerical simulation. As shown in Figure 11b, the maximum temperature was 41.23 °C, with an error of 2%. This represents a drop of 11.21 °C, compared with that under original packaging form. According to calculations using Equations (6) and (7), the light-emitting power rose from 50.582 W to 52.759 W, with an increase of 1.8 W, and the light emitting efficiency increased from 91.8% to 95.39%.

## 5. Analysis on Applicability of Optimized MCHS to the LD with Higher Power

**Figure 12:**
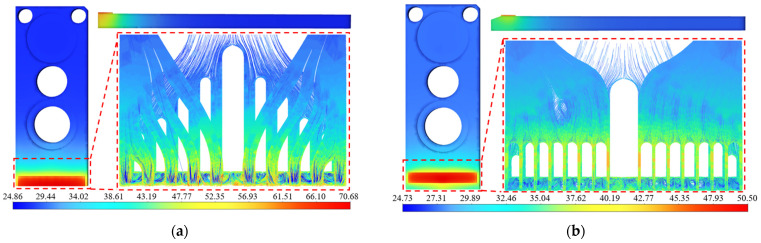
Thermal analysis of 100 W rod package optimization. (**a**) The maximum temperature in the original package is 70.68 °C. (**b**) The maximum temperature of the package after optimization is 50.50 °C.

Furthermore, the research group chose an LD bar with a continuous emission power of up to 100 W at 808 nm in the laboratory to explore the applicability of the optimized packaging form to the heat dissipation of higher-power LD bars. The thickness of the LD bars was 125 μm, the cavity length was 1500 μm, and the photoelectric conversion efficiency was 55%. The simulation results are shown in Figure 12.

During production and application, an LD with a power degradation of more than 10% was generally considered as failure, which was based on the product manual of the LD chips. When 100 W bars were packaged in the original form, the maximum temperature of the simulation test was 70.67 °C. According to Equations (6) and (7), the calculated light emitting power was 87.021 W, and the power degradation was 12.979%, which exceeds the failure criteria of 10%. When the optimized packaging form was adopted, the maximum temperature was 50.50 °C, the light emitting power was 92.592 W, and the power degradation was 7.408%, which is less than the 10% threshold, so it was qualified. These results demonstrate that the optimized packaging form can meet the heat-dissipation requirements of the HPLD with a continuous emission power of up to 100 W, with universal applicability. The actual packaging trial showed that the LD can operate stably, with good performance.

## 6. Conclusions

In this study, the heat-dissipation structure of the HPLD was optimized based on the space environment. In brief, a fluid–solid coupled conjugate heat transfer model was constructed for numerical simulation, and the influence of the bar packaging position on the heat-dissipation performance of the MCHS was studied. In addition, the relationship between the MCHS’s structural parameters and the maximum laser temperature was obtained by a single factor experiment and an orthogonal experiment. Moreover, the optimization of key structural parameters was accomplished by the combination of a neural network algorithm and a genetic algorithm. The following conclusions are drawn:

(1) The rearward shift of the LD bar packaging position could significantly improve the heat-dissipation performance of the MCHS. In the case of a bar rearward shift distance of 2 mm, optimal heat-dissipation performance was achieved, resulting in a maximum temperature decline of 6.75 °C and a thermal resistance drop of 24.6%. If the bar rearward shift distance was over 2 mm, the heat-dissipation performance decreased.
(2) The structure of the MCHS was optimized, and a single-factor experiment and an orthogonal experiment were carried out to develop the polynomial regression model between key structural parameters of the MCHS and the maximum temperature, followed by verification of the goodness-of-fit and reliability of the model. Additionally, by virtue of a neural network algorithm combined with GA, the optimal key structural parameter combination of the MCHS was determined as W1 = 0.5 mm, W2 = 0.1 mm, and L1 = 1.219 mm. The heat-dissipation performance of the optimized MCHS was obviously enhanced, with the maximum temperature decreasing from 52.44 °C to 41.23 °C.
(3) A comparative study was performed on bars with continuous emission power of up to 100 W before and after optimization. The results revealed that the packaging scheme before optimization failed to meet the heat-dissipation requirements, whereas the optimized packaging structure satisfied the heat-dissipation requirements of 100 W bars.

In the present study, the heat dissipation of the LD in a space environment is optimized, and a scheme enhancing heat conduction efficiency and heat-dissipation performance is put forward. The results revealed that the optimized packaging form displays excellent overall performance, but it also has the shortcoming of difficult processing. The results provide a valuable reference for studies on heat-dissipation performance and thermal reliability of the HPLD with microchannel water cooling. For the HPLD based on the MCHS packaging, there is still a room for further improvement in overall heat dissipation-performance.

## Figures and Tables

**Table 1:** Parameter values of thermal calculation in the paper.

Parameter	Numerical Value	Unit
Output power ($P_{out}$)	55.31	W
Electro-optical conversion efficiency (η)	58.74	%
Electric current (*I*)	50	A
Reference temperature ($T_{Ref}$)	25	°C
Slope efficiency ($\eta_{Ref}$)	1.21	W/A
Characteristic temperature ($T_{0}$)	400	K
Threshold current ($I_{Ref}$)	4.4	A

**Table 2:** Level table of influencing factors.

Level	W1/mm	W2/mm	L1/mm
−1	0.1	0.1	0
0	0.3	0.3	2
1	0.5	0.5	4

**Table 3:** Orthogonal test results.

Group	W1/mm	W2/mm	L1/mm	Tmax/°C
1	0.3	0.3	2	44.21
2	0.5	0.5	2	47.19
3	0.3	0.5	0	53.18
4	0.5	0.3	4	45.27
5	0.1	0.3	4	45.54
6	0.3	0.3	2	44.71
7	0.3	0.3	2	44.76
8	0.1	0.1	2	41.09
9	0.5	0.3	0	48.85
10	0.3	0.3	2	44.74
11	0.3	0.1	4	40.84
12	0.5	0.1	2	41.14
13	0.1	0.3	0	52.05
14	0.3	0.5	4	47.7
15	0.1	0.5	2	47.82
16	0.3	0.1	0	45.5
17	0.3	0.3	2	44.96

**Table 4:** Randomized data training results and test results.

	Sample 1	Sample 2	Sample 3	Sample 4	Sample 5	Sample 6	Sample 7	Sample 8
W1/mm	0.3	0.5	0.1	0.3	0.1	0.5	0.3	0.1
W2/mm	0.5	0.5	0.3	0.1	0.3	0.3	0.5	0.1
L1/mm	2	2	4	4	0	0	4	2
R1/°C	46.856	47.4911	45.0608	41.0647	52.4636	48.8154	47.1176	40.9857
R2/°C	47.12	47.19	45.54	40.84	52.05	48.85	47.70	41.09

## Data Availability

The original contributions presented in this study are included in the article. Further inquiries can be directed to the corresponding authors.
